# Supplementary material for: Mathematics teachers’ reflective thinking: Level of understanding and implementation in their professional practices
Source: PLoS One. 2021 Oct 7;16(10):e0258149. doi: 10.1371/journal.pone.0258149 (PMC8496853; doi:10.1371/journal.pone.0258149)
Supplement: Attachment — Submitted filename: Response to Reviewers.docx [file pone.0258149.s001.docx]

**Rebuttal letter**

PLOS ONE Decision: Revision required [PONE-D-21-16098] - [EMID:439b9ab96b8daaf1]‏‏

Dear José Gutiérrez-Pérez
Academic Editor
PLOS ONE

I hope you are doing very well

Here is a rebuttal letter including responses to the editor and the review’s comments. I have taken care of almost all comment and concerns raised by the reviewers and the editor. All responses are highlighted in green color.

Dear Dr. Aldahmash, 

Thank you for submitting your manuscript to PLOS ONE. After careful consideration, we feel that it has merit but does not fully meet PLOS ONE’s publication criteria as it currently stands. Therefore, we invite you to submit a revised version of the manuscript that addresses the points raised during the review process.


Please submit your revised manuscript by 07/29/2021. If you will need more time than this to complete your revisions, please reply to this message or contact the journal office at [plosone@plos.org](mailto:plosone@plos.org). When you're ready to submit your revision, log on to <https://www.editorialmanager.com/pone/> and select the 'Submissions Needing Revision' folder to locate your manuscript file.

Please include the following items when submitting your revised manuscript:

- A rebuttal letter that responds to each point raised by the academic editor and reviewer(s). You should upload this letter as a separate file labeled 'Response to Reviewers'.
- A marked-up copy of your manuscript that highlights changes made to the original version. You should upload this as a separate file labeled 'Revised Manuscript with Track Changes'.
- An unmarked version of your revised paper without tracked changes. You should upload this as a separate file labeled 'Manuscript'.

If you would like to make changes to your financial disclosure, please include your updated statement in your cover letter. Guidelines for resubmitting your figure files are available below the reviewer comments at the end of this letter.

**Reply: Regarding the financial status, I will pay the fee from my own budget. However, if the fees are high, I hope that a percent of the due money could be waved due to the fact that the fund is not high enough and that the first and third authors are Yemeni nationals, while the second author is a Saudi national.**

If applicable, we recommend that you deposit your laboratory protocols in [protocols.io](http://protocols.io/) to enhance the reproducibility of your results. Protocols.io assigns your protocol its own identifier (DOI) so that it can be cited independently in the future. For instructions see: <http://journals.plos.org/plosone/s/submission-guidelines#loc-laboratory-protocols>. Additionally, PLOS ONE offers an option for publishing peer-reviewed Lab Protocol articles, which describe protocols hosted on [protocols.io](http://protocols.io/). Read more information on sharing protocols at <https://plos.org/protocols?utm_medium=editorial-email&utm_source=authorletters&utm_campaign=protocols>.

**Reply: I think these protocols do not apply to our article, because it is an educational type research.**

We look forward to receiving your revised manuscript.

Kind regards,

José Gutiérrez-Pérez
Academic Editor
PLOS ONE

Journal requirements:

When submitting your revision, we need you to address these additional requirements.

1. Please ensure that your manuscript meets PLOS ONE's style requirements, including those for file naming. The PLOS ONE style templates can be found at

<https://journals.plos.org/plosone/s/file?id=wjVg/PLOSOne_formatting_sample_main_body.pdf> and

https://journals.plos.org/plosone/s/file?id=ba62/PLOSOne_formatting_sample_title_authors_affiliations.pdf

**Reply: requirements were followed.**

2. Please provide additional details regarding participant consent. In the ethics statement in the Methods and online submission information, please ensure that you have specified (1) whether consent was informed and (2) what type you obtained (for instance, written or verbal, and if verbal, how it was documented and witnessed). If your study included minors, state whether you obtained consent from parents or guardians. If the need for consent was waived by the ethics committee, please include this information.

**Reply: the ethical committee sent us an email regarding their consent to apply the reflective thinking questionnaire on mathematics teachers.**

**I have included the following statements:**

- **The participants in this study sample were informed of the study objectives at the beginning, in the instruction part, of the questionnaire. They were also informed that participation was not obligatory, and that they did not have to write their names or identity. In addition, this study had been approved by the Research Ethics Committee of Deanship of Scientific Research, King Saud University.**
- **We obtained informed written consent from the Research Ethics Committee of Deanship of Scientific Research, King Saud University via email**
- **I ensure that all the authors mentioned in the manuscript have agreed for authorship, read and approved the manuscript, and given consent for submission and subsequent publication of the manuscript.**

If you are reporting a retrospective study of medical records or archived samples, please ensure that you have discussed whether all data were fully anonymized before you accessed them and/or whether the IRB or ethics committee waived the requirement for informed consent. If patients provided informed written consent to have data from their medical records used in research, please include this information.

**Reply: Not applicable**

3. Thank you for stating the following in the Acknowledgments Section of your manuscript:

"We thank the Excellent Research Center of Science and Mathematics Education, ECSME for
their support in conducting this research"

We note that you have provided funding information that is not currently declared in your Funding Statement. However, funding information should not appear in the Acknowledgments section or other areas of your manuscript. We will only publish funding information present in the Funding Statement section of the online submission form.

**Reply: Ok**

Please remove any funding-related text from the manuscript and let us know how you would like to update your Funding Statement. Currently, your Funding Statement reads as follows:

"This research was supported by the Deanship of Scientific Research, Research at King Saud University, Group no. (RG-1440-123) "

**Reply: OK**

Please include your amended statements within your cover letter; we will change the online submission form on your behalf.

**Reply: I am and the third author are Yemen nationals, while the second author is a Saudi national. Therefore, I wish if you could reduce the open publishing fee.**

**Comments to the Author**

1. Is the manuscript technically sound, and do the data support the conclusions?

The manuscript must describe a technically sound piece of scientific research with data that supports the conclusions. Experiments must have been conducted rigorously, with appropriate controls, replication, and sample sizes. The conclusions must be drawn appropriately based on the data presented.

Partly

2. Has the statistical analysis been performed appropriately and rigorously?

Parthly

3. Have the authors made all data underlying the findings in their manuscript fully available?

The [PLOS Data policy](http://www.plosone.org/static/policies.action#sharing) requires authors to make all data underlying the findings described in their manuscript fully available without restriction, with rare exception (please refer to the Data Availability Statement in the manuscript PDF file). The data should be provided as part of the manuscript or its supporting information, or deposited to a public repository. For example, in addition to summary statistics, the data points behind means, medians and variance measures should be available. If there are restrictions on publicly sharing data—e.g. participant privacy or use of data from a third party—those must be specified.

**Reply: OK**

Patly

4. Is the manuscript presented in an intelligible fashion and written in standard English?

PLOS ONE does not copyedit accepted manuscripts, so the language in submitted articles must be clear, correct, and unambiguous. Any typographical or grammatical errors should be corrected at revision, so please note any specific errors here.

Partly

5. Review Comments to the Author

Please use the space provided to explain your answers to the questions above. You may also include additional comments for the author, including concerns about dual publication, research ethics, or publication ethics. (Please upload your review as an attachment if it exceeds 20,000 characters)

Reviewer #1: Measuring teachers’ reflective thinking on teaching practices during mathematics classroom is important to the teacher development programs. This study’s main goal is to examine the math teachers’ skills in the context of how they are applied to their understanding of their teaching practices. The adequate instrument and proper statistical analysis were performed appropriately and rigorously. The authors concluded that the teachers’ practices concerning all the themes of reflective thinking addressed by the questionnaire, especially their ability to self-assess, fluctuated around the introductory, intermediate, and advanced levels. The authors expect to strengthen the rationale motivating further research exploring Saudi Arabian math teachers’. Because of this, the current study is on a topic of relevance and general interest to the readers of the journal. It is suggested to publish. Please carefully proof-read spell check to eliminate typing and grammatical errors before its acceptance.

**Reply: I have taken care of all comments and concerns raised by the first reviewer.**

Reviewer #2: The paper lacks a lot of important details on teaching mathematics, why is it important for mathematics teachers conduct reflective thinking in teaching mathematics. The literature review was done in a brief manner. It is suggested the authors focus on addressing the literature and theoretical gaps before presenting the problem statement.

**Reply: I have added paragraphs to explain why is it important for mathematics teachers conduct reflective thinking in teaching mathematics. (p.3 )**

There was no discussion of the instruments available to measure mathematics teachers reflective thinking and why the 1997's instrument was used in this study.

**Reply: I have added a paragraph discussing the instruments available to measure reflective thinking among teachers in general and mathematics teachers in particular, and explaining why the 1997's instrument was used in this study. (p.6)**

Initially I thought the research only collected quantitative data as there was no explanation of qualitative data collection in the research method. Suddenly, there was an interview data after the quantitative data was presented. I am not sure the research design of this study. There was also no discussion on how the interview data was analysed.

**Reply: an explanation of qualitative data collection was added in the research method (p.8)**

The discussion was not linked to the literature review of the paper hence lacks of significant contribution towards the body of knowledge in teacher development.

**Reply: discussion was linked to the literature review to ensure the significant contribution towards the body of knowledge in teacher development (p. 19-20)**

There are also many unforgiven grammatical and spelling mistakes.

**Reply: grammatical and spelling check has been made throughout the manuscript**.

Hence, it is proposed that this paper to be either given a major revision or rejected from publication in a prestigious journal as PLOS.

**Reply: Major revision has been implemented.**

Reviewer #3: Here are my comments to the paper:
1. The first comment is that there are a lot of citations whose sources are not in the reference list:
- Duban, Yanpar, & Yelken, 2010
- Dymoke and Harrison (2008)
- (Cohen et al., 2000)
- Branch & Paranjape (2002)
- Halpern (1996)
- Boody (2008)
- Riddell (2007)
- Brookfield (1988)
- Shermis (1999)

**Reply: All references have been carefully checked whether in the text of the in the list (see the tracked version of the manuscript)**

2. Check the following references which are not cited in the text:
-Chen, D., & Stroup, W. (1993). General system theory: Toward a conceptual framework for
science and technology education for all. Journal of Science Education and
Technology, 2(3), 447-459.
-Bell, P., & Linn, M. C. (2000). Scientific arguments as learning artifacts: Designing for
learning from the web with KIE. International Journal of Science Education, 22(8), 797–
817. <https://doi.org/10.1080/095006900412284>
- Hood, S. (2008). Linguistics and education: An International Research Journal, 19(4), 351–
365.

**Reply: All references have been carefully checked whether in the text of the in the list (see the tracked version of the manuscript)**

3. Theoretically and methodologically the work is well founded.

**Reply: Don**

4. My main concerns are in the discussion:
- How do authors justify the predominance of the level "Introductory, intermediate and advanced"in the light of Tables 1, 2 and 3. Please elaborate more on this issue.

- On paragraph "Concerning the third theme,..., (Saavedra & Opfer, 2012), there is a repetition of categories related to the second theme.

**Reply: I have made the correction of this part (P.13)**

5. Check out the following mistakes in the text:

- (paragraph Dewey (1933) and Schon (1983)...): "Reflection represents the highest level of the eight categories of argumentation", Which are these eight categories?
- (Ennis, 1996; 1987)--> (Ennis, 1987, 1996)
- Related studies have been don --> Related studies have been done
- For example, Fariba Mirzaeia, Fatin Aliah, ...-> Mirzaei, Phang and Kashefi (2014)
- Choy, & Oo, (2012)--> Choy and Oo (2012)
- "teachers neglect the practice these four"--> "Teachers neglect the practice of these four..."
- Oo, Pou & Choy, Chee (2010)--> Oo and Choy (2010)
- Fariba et al (2013)->Mirzaei et al. (2014)
- (Fernandez, Karsenti & Charlin, 2014)-> (Nguyen et al., 2014)
- School mathematics teachers on-duty --> school mathematics teachers on duty.
- Strongly agree: From 4:21 to 5:00--> From 4.21 to 5.00, check this throughout the text!!!
- "were made to translation of the statements"--> were made to the translation of the statements
- "Cronbach's Alpha's coefficient-> Cronbach's Alpha Coefficient
- "ability to self-asses"--> ability to self-assess
- Schon (1987) added--> 1983 or 1987, check the year of the reference.
- (Allinder, 1994)--> (Allinder, 2009), check the year of the reference.
- Explain what PD programs and CPD providers stand for.

**Reply: I have made the required correction of all mistakes. (all citations have been changed according to the journal system)**

Reviewer #4: This paper reports the results of a large survey about mathematics teachers’ reflective thinking, where the questionnaire used was translated from English to Arabic and used in Saudi Arabia. I think that the outcomes might be of interest to a practitioner journal in that region. But, as presented here, the work does not make a contribution to knowledge at the level that would be expected in an international research journal. Below, I explain why, and also add some minor notes that the authors might wish to take into account if they decide to resubmit to this.

**Reply: I have taken care of most of the concerns raised by the 4rth reviewer.**

The main issue I see is that the paper’s main results are simply the average scores across the participants for each of the 33 self-report questionnaire items. These, on their own, do not tell us much. They are not really comparable with one another – what would it mean to say that teachers are more or less inclined to observe their performance than they are aware of how one learns? And, here, they are not related to anything else – for instance, we do not find out whether more experienced teachers tend to be more reflective (or to score higher on some subscale), or whether mathematics teachers are more or less reflective than teachers in other subjects, or whether more reflective teachers get better results or more participation from their students. Perhaps the authors have the data to link the scores to another construct like one of these. If so, that would strengthen the paper considerably.

**Reply: an ANOVA test was done to see if there are differences in teachers’ reflective thinking related to experiences, in addition to another table that sum-up the results in a more clearer way (see p. 14-15)**

To list some more minor issues:
• Some aspects of the introductory sections use strong language (“cannot”, “essential” etc.), which sound like the authors are making an absolute judgement about what is right or necessary rather than relying on research evidence.

**Reply: I have changed many wordings to avoid making an absolute judgement.**

• There is more than one list of constructs related to reflective thinking (Choy & Oo, Fariba et al.) but the authors do not explain whether and how these are linked.

**Reply: I have taken care of this issue**

• For an international journal, some information on the Saudi Arabian context will be needed, e.g. what age are students in high school, do all teachers teach in the same types of school, etc. This needn’t be long, but would help international readers to compare with their own contexts.

**Reply: age of students and characteristics of schools have been added (p.6)**

• The abstract says that the survey involved 602 teachers, but apparently only 322 responded.

**Reply: sample # have been corrected**

• There are some numbers unnecessarily in brackets in the description of the instrument.

**Reply: Numbers are necessary in the description of the instruments and their items (p.6 )**

• Abbreviations and capitalisation are used inconsistently, e.g. RTTW vs. RTATQ, capitalising Excellent Centre or not.

**Reply: I capitalized all**

• In reading about the scale, I was initially confused by the referencing and thought Williams & Burden had constructed it.

**Reply: Williams & Burden had constructed the instruments and was modified by Choy & Oo (2012) (p.6 )**

• I thought the ranges (strongly agree from 4.21 to 5.00 etc.) confusing and unnecessary. If I know there is a scale from 1 to 5, I can interpret what an average of 4.6 means. Also, I think that “weighted average” is misused here, and the authors just mean “mean”.

**Reply: the weighted averages have been replaced by means**

• The authors say that their main goal was to discover whether there was a statistically significant difference in mathematics teachers’ practices of reflective thinking, but they do not report any statistical tests about differences so this is not addressed.

**Reply: I have added table 5 to present statistical differences according to experiences (p. 15)**

• I was confused by the fact that the I, In and A items appear in different orders in different sections of the table. This makes it harder to compare across them.

**Reply: I have changed the orders (p 9-10)**

• There is no report of the sampling strategy or analysis process for the qualitative interviews, and these are not systematically related to the quantitative da

**Reply: the open ended question were given to all samples, because they were combined with the survey (p.6 )**

[NOTE: If reviewer comments were submitted as an attachment file, they will be attached to this email and accessible via the submission site. Please log into your account, locate the manuscript record, and check for the action link "View Attachments". If this link does not appear, there are no attachment files.]

While revising your submission, please upload your figure files to the Preflight Analysis and Conversion Engine (PACE) digital diagnostic tool, <https://pacev2.apexcovantage.com/>. PACE helps ensure that figures meet PLOS requirements. To use PACE, you must first register as a user. Registration is free. Then, login and navigate to the UPLOAD tab, where you will find detailed instructions on how to use the tool. If you encounter any issues or have any questions when using PACE, please email PLOS at [figures@plos.org](mailto:figures@plos.org). Please note that Supporting Information files do not need this step.
