# Supplementary material for: Mathematics teachers’ reflective thinking: Level of understanding and implementation in their professional practices
Source: PLoS One. 2021 Oct 7;16(10):e0258149. doi: 10.1371/journal.pone.0258149 (PMC8496853; doi:10.1371/journal.pone.0258149)
Supplement: Attachment — Submitted filename: Rebuttal Litter.docx [file pone.0258149.s002.docx]

**Rebuttal Litter2**

**Journal Requirements:**

Please review your reference list to ensure that it is complete and correct. If you have cited papers that have been retracted, please include the rationale for doing so in the manuscript text, or remove these references and replace them with relevant current references. Any changes to the reference list should be mentioned in the rebuttal letter that accompanies your revised manuscript. If you need to cite a retracted article, indicate the article’s retracted status in the References list and also include a citation and full reference for the retraction notice.

**Answer:**

Most of the references were had been reviewed and reordered.

The retracted references were not cited in the text body, and were irrelevant to the paper.

all references were reordered according to their original sources to ensure that it is complete and correct (see the markup copy in which each reference number was aligned with its original source and was written as a merging comment. (P. 17-19)

**Comments to the Author**

Please use the space provided to explain your answers to the questions above. You may also include additional comments for the author, including concerns about dual publication, research ethics, or publication ethics. (Please upload your review as an attachment if it exceeds 20,000 characters)

In general, I observe an improvement of the paper. My main concern is related to the interpretation of Table 1, 2, 3 and 4.

Note that in table 1, item #1, the weighted mean is 3.02, rather than 3.10

**Answer:**

weighted mean in table1 had been changed from 3.10 to 3.02

First, in the following paragraph:

The teachers’ ability to "make judgements" was most prevalent at the introductory level, where the highest weighted average was 4.42, indicating that the introductory-level teachers "strongly agreed"
with this item.

The value 4.42 is related to the advanced level, not the introductory level as authors stated.

**Answer:**

- According to the highest mean (4.4), the level of the sub-theme “make judgements“ was changed to advanced rather than intermediated or introductory (Table1).
- According to the highest mean (4.60) the level of the sub-theme “ability to observe their performance" was changed to advanced rather than intermediated (Table1). introductory
- According to the highest mean (4.04), the level of the sub-theme “ability to use feedback and evidence " “was changed to intermediated (Table1).
- According to the highest mean(4.41), the level of the sub-theme “ability to *Finding and analyzing patterns* " “ was changed to advanced (Table1).
- The level for the hole theme “ ability to self-assess is Advanced because the highest mean was 4.37, whereas the mean of the intermediate level is 4.29, and the introductory level was 2.81

  Second, in the following paragraph:

  In short, math teachers’ ability to "observe their performance" was most prevalent at the
  introductory level, their ability to "use feedback and evidence" was most prevalent at both the intermediate and advanced levels, their ability to "find and analyze patterns" was most prevalent at the advanced level, and their ability to "make judgments" was most prevalent at the introductory level. As a result, math teachers’ ability to "self-assess" was found predominantly at the intermediate level, where
  the value for the weighted average was 3.85.

  Check that the highest weighted mean in "observing the performance" is 4.60 which corresponds to Advanced level, and in "make judgements" is 4.42 which corresponds to Advanced level, Where does the value 3.85 come from? I think that authors made the average of each level (I, In and A) and then chosen he highest value:
  - For example, from table 1. Level I has an average of (3.02+2.78+4.39+2.75)/4=3.24 ; Level In has an average of 3.98; and Level A has an average of 4.37
  So the proper value is 3.98

**Answer:**

The level of the sub-theme “ability to observe their performance" “was changed to advanced rather than intermediated or introductory

The level of the sub-theme “ability to use feedback and evidence " “was changed to advanced rather than intermediated or introductory

The total mean for the intermediated level of the first theme was changed to 3.98

The level for the hole theme is Advanced because the highest mean was 4.37, whereas the mean of the intermediate level is 4.29, and the introductory level was 2.81 (P. 6-7)

The total of the averages for each level were calculated and placed under the table

Third, in the following paragraph:
This theme consisted of three sub-themes, as shown in Table 2. The results for the first sub-theme, "concepts and misconceptions," indicated that math teachers’ awareness of concepts and misconceptions was most prominently observed between the intermediate and advanced levels, where the mean for the frequencies were 4.14 and 4.28, respectively. Meanwhile, the results of the second sub-theme, "knowledge construction," indicated that the teachers’ ability to construct knowledge also concentrated around the intermediate and advanced levels. Similarly, the results for the third sub-theme, "metacognition," showed that teachers’ belief in their metacognitive ability was most prevalent at the intermediate and advanced levels; the intermediate weighted average for the frequency of this level was
4.28.

Where does value 4.28 come from? By calculating the average of each level (N=3, sub-themes), Introductory level has an average of 2.81; Intermediate level (4.286..., so 4.29); Advanced level (4.37). As done before in table 1, authors may conclude that 4.37 is that is an advanced level predominance in theme "teachers' awareness of how one learns" (p. 7-8)

So in paragraph:
The means for the categories of the theme, "awareness of how one learns," indicated that teachers’ abilities to identify "concepts and misconceptions," "knowledge construction," and "metacognition" are predominantly at the [[[intermediate level]]. The proper level is advanced (4.37).

**Answer:**

The proper level was changed to advanced (4.37) (p. 7-8)

Related to table 4, I do not understand the statement and ask the authors to explain a little more. "All the values of the means were found to center around the "agree" response, which the grading rules indicated was between 3.41 and 4.2" (3:41 and 4:2 are typos).

**Answer:**

The level of the sub-theme “*Concepts and misconceptions* “ is Advanced because the highest mean value is 4.28

The level of the sub-theme “*Knowledge construction* “ is Advanced because the highest mean value is 4.48

The level of the sub-theme “*Metacognition* “ is Intermediate because the highest mean value is 4.41

The level for the hole theme is Advanced because the highest mean was 4.37, whereas the mean of the intermediate level is 4.29, and the introductory level was 2.81 . (p. 10-11)

The total of the averages for each level were calculated and placed under the table

In table 5, Subtotal cells includes the average of each level for each skill. The total cells are the average of the four subtotal values. This has to be explicited.

**Answer:**

Percentages have been added to table5 to make the interpretation clearer, and to support the results reported earlier. In addition, some changes were made to the interpretation of the data to be explicated. (p. 11)

The total of the averages for each level were calculated and placed under the table

The important thing is that authors should keep the same way to discuss the results from the four themes.
Finally, I have detected a mistake in the "Discussion" section (Studies [49, 9; 4], It should be Studies [4, 9, 49]

**Answer:**

Parts of the discussion has been change to be aligned with the changes made to the results parts (p. 13-15)

The order of the studies [49, 9; 4] has been changed to [4, 9, 49]. (p. 13-15)

Congratulations to authors.

**Great thanks to the reviewers, especially who made the second revision, who made thorough reading, and was very keen in his revision.**

[NOTE: If reviewer comments were submitted as an attachment file, they will be attached to this email and accessible via the submission site. Please log into your account, locate the manuscript record, and check for the action link "View Attachments". If this link does not appear, there are no attachment files.]

While revising your submission, please upload your figure files to the Preflight Analysis and Conversion Engine (PACE) digital diagnostic tool, <https://pacev2.apexcovantage.com/>. PACE helps ensure that figures meet PLOS requirements. To use PACE, you must first register as a user. Registration is free. Then, login and navigate to the UPLOAD tab, where you will find detailed instructions on how to use the tool. If you encounter any issues or have any questions when using PACE, please email PLOS at [figures@plos.org](mailto:figures@plos.org). Please note that Supporting Information files do not need this step.
